# P-144. The Introduction of Antimicrobial Prescription Guidance and Patient Education for a Medical Brigade to Honduras

**DOI:** 10.1093/ofid/ofae631.349

**Published:** 2025-01-29

**Authors:** Moira Anderson, Gabriella Zerbini, Danielle G Abdallah, Amy M James

**Affiliations:** The Ohio State University College of Medicine, Columbus, Ohio; The Ohio State University College of Pharmacy, Columbus, Ohio; The Ohio State University College of Medicine, Columbus, Ohio; The Ohio State University, Columbus, Ohio

## Abstract

**Background:**

Antimicrobial resistance is a global problem that requires active interventions to combat. Misusage of antimicrobials contributes to the development of multi-drug resistant organisms that can be fatal. Many can be purchased over the counter in countries outside of the US, and medical brigades often provide a source of these drugs. PODEMOS is a medical brigade partnered with The Ohio State University College of Medicine and travels to Honduras twice yearly. Since this brigade prescribes antimicrobials for a variety of disease states, this project aimed to create guidelines on antimicrobial usage and community education materials to ensure that PODEMOS is not contributing to further resistance.

Patient Education Antimicrobial Resistance

**Figure:**
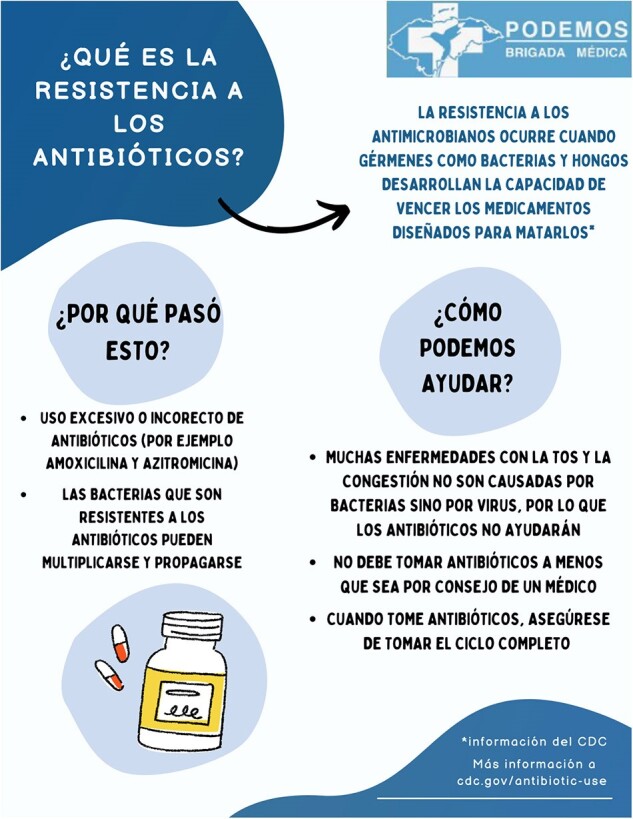

This flyer was distributed to patients on the PODEMOS medical brigade to educate the community on antimicrobial resistance.

**Methods:**

First, current literature on antimicrobial resistance was reviewed to understand the scope of the problem in Central America. Data was collected on antimicrobial consumption and community education within Honduras and surrounding area. Secondly, common infectious diseases encountered during the PODEMOS medical brigades were identified and the current drug formulary was reviewed in comparison to current treatment guidelines on UpToDate.

Medical Brigade Treatment Guidelines

**Figure:**
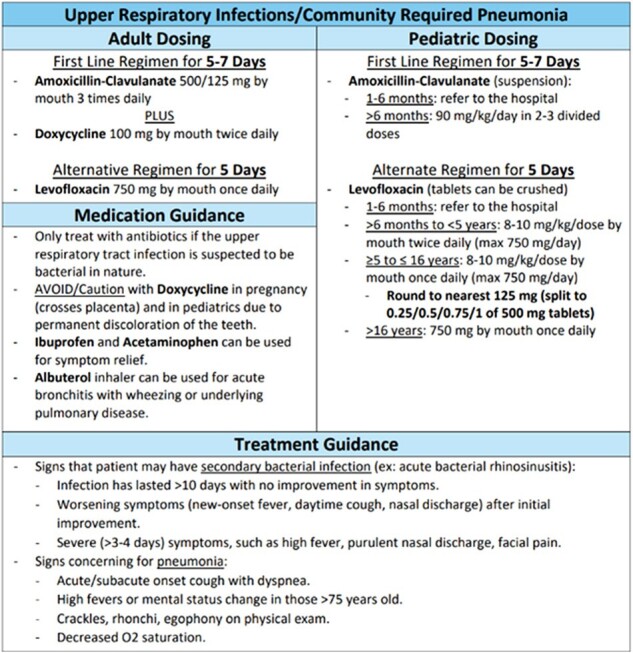

The image shows a portion of the guidelines that were created for PODEMOS medical brigades, with this example being for upper respiratory infections.

**Results:**

Results of our initial literature review indicated that there is no current standard of tracking antimicrobial usage in Honduras. There is also limited awareness and education on antimicrobial resistance. There were 16 common infectious disease states encountered on PODEMOS brigades which were used as basis for creating guidelines for students and providers on future brigades. These included information on clinical diagnosis, treatment protocols, and medication safety. Also, the drug formulary for PODEMOS was updated to include two antimicrobials that will lessen contribution to drug resistance. Lastly, a flyer was created with information to educate the community on antimicrobial resistance.

**Conclusion:**

Guidance for the proper usage of antimicrobials is paramount in combating drug resistance. By creating treatment protocols and patient education materials, this project aimed to prevent contribution to antimicrobial resistance and improve the care of patients on future PODEMOS medical brigades. Prospectively, data will be collected on usage of these guidelines and patient understanding of antimicrobial resistance.

**Disclosures:**

**All Authors**: No reported disclosures

